# Study protocol: Whole genome sequencing Implementation in standard Diagnostics for Every cancer patient (WIDE)

**DOI:** 10.1186/s12920-020-00814-w

**Published:** 2020-11-10

**Authors:** Kris G. Samsom, Linda J. W. Bosch, Luuk J. Schipper, Paul Roepman, Ewart de Bruijn, Louisa R. Hoes, Immy Riethorst, Lieke Schoenmaker, Lizet E. van der Kolk, Valesca P. Retèl, Geert W. J. Frederix, Tineke E. Buffart, Jacobus J. M. van der Hoeven, Emile E. Voest, Edwin Cuppen, Kim Monkhorst, Gerrit A. Meijer

**Affiliations:** 1 Department of Pathology, Netherlands Cancer Institute, Amsterdam, The Netherlands; 2 Department of Molecular Oncology, Netherlands Cancer Institute, Amsterdam, The Netherlands; 3 Hartwig Medical Foundation, Amsterdam, The Netherlands; 4 Family Cancer Clinic, Netherlands Cancer Institute, Amsterdam, The Netherlands; 5 Department of Gastrointestinal Oncology, Netherlands Cancer Institute, Amsterdam, The Netherlands; 6 Center for Molecular Medicine and Oncode Institute, University Medical Center Utrecht, Utrecht, The Netherlands

**Keywords:** Cancer, Diagnostics, Whole genome sequencing, Biomarker, Personalized medicine

## Abstract

**Background:**

‘Precision oncology’ can ensure the best suitable treatment at the right time by tailoring treatment towards individual patient and comprehensive tumour characteristics. In current molecular pathology, diagnostic tests which are part of the standard of care (SOC) only cover a limited part of the spectrum of genomic changes, and often are performed in an iterative way. This occurs at the expense of valuable patient time, available tissue sample, and interferes with ‘first time right’ treatment decisions. Whole Genome Sequencing (WGS) captures a near complete view of genomic characteristics of a tumour in a single test. Moreover, WGS facilitates faster implementation of new treatment relevant biomarkers. At present, WGS mainly has been applied in study settings, but its performance in a routine diagnostic setting remains to be evaluated. The WIDE study aims to investigate the feasibility and validity of WGS-based diagnostics in clinical practice.

**Methods:**

1200 consecutive patients in a single comprehensive cancer centre with (suspicion of) a metastasized solid tumour will be enrolled with the intention to analyse tumour tissue with WGS, in parallel to SOC diagnostics. Primary endpoints are (1) feasibility of implementation of WGS-based diagnostics into routine clinical care and (2) clinical validation of WGS by comparing identification of treatment-relevant variants between WGS and SOC molecular diagnostics. Secondary endpoints entail (1) added clinical value in terms of additional treatment options and (2) cost-effectiveness of WGS compared to SOC diagnostics through a Health Technology Assessment (HTA) analysis. Furthermore, the (3) perceived impact of WGS-based diagnostics on clinical decision making will be evaluated through questionnaires. The number of patients included in (experimental) therapies initiated based on SOC or WGS diagnostics will be reported with at least 3 months follow-up. The clinical efficacy is beyond the scope of WIDE. Key performance indicators will be evaluated after every 200 patients enrolled, and procedures optimized accordingly, to continuously improve the diagnostic performance of WGS in a routine clinical setting.

**Discussion:**

WIDE will yield the optimal conditions under which WGS can be implemented in a routine molecular diagnostics setting and establish the position of WGS compared to SOC diagnostics in routine clinical care.

## Background

Matching the best possible treatment with specific characteristics of a patient’s tumour is the aim and challenge of ‘precision oncology’. Development of a wide range of targeted drugs and their associated biomarkers has led to a large variety of diagnostic platforms being used in standard of care (SOC) molecular diagnostics. These include targeted next generation sequencing (NGS) panels, RNA-based NGS fusion analysis, Sanger sequencing, reverse transcription polymerase chain reaction (RT-PCR), fluorescence in situ hybridization (FISH) and immunohistochemistry (IHC). Each of these tests covers only a single or limited part of the spectrum of relevant genomic changes. As a consequence, multiple tests are often performed iteratively in clinical practice, starting with profiling of the most prevalent biomarkers, followed by less prevalent biomarkers if necessary. However, this occurs at the expense of valuable patient time, tissue sample, and this strategy may interfere with making ‘first time right’ treatment decisions.

Hence, there is a need for an affordable comprehensive diagnostic approach, which optimally uses the available tumour tissue, can report within an acceptable time frame and is able to keep up with the pace of the rapidly changing current oncological landscape with respect to new treatment options and associated biomarkers. This rapidly changing oncological landscape poses a major challenge for standard molecular diagnostics since there is an increasing need for broader molecular testing of a growing patient population. Whole Genome Sequencing (WGS) captures a near complete overview of genomic characteristics of a tumour in one test, using a relatively low amount of tumour material. WGS thereby allows the streamlining of laboratory logistics—and thus patient care—with one comprehensive test. Furthermore, due to the steadily decreasing DNA sequencing costs over the past decades, WGS is becoming an attractive alternative to standard molecular diagnostics. Additional potential benefits of WGS include the option to test biomarkers for experimental clinical trials and the development of new biomarkers including complex biomarkers such as signatures. Most importantly, the potential therapeutic benefit of WGS for patients with metastatic cancer has clearly been documented. In recent years, the Hartwig Medical Foundation (HMF), in collaboration with the Center for Personalized Cancer Treatment (CPCT), has performed an in-depth retrospective pan-cancer WGS analysis on metastatic tumour and normal genome analysis of the first 2500 patients. Based on an analysis of a subset of these patients (n = 1480), at least one ‘clinically actionable’ target could be identified for up to 62% of patients [1]. In 31% of the subset, a match was found for an actionable target and a registered and approved therapy. In 13% of cases, this match was an off-label indication (approved for another tumour type) for a target, which most likely would not have been detected using common panel-based NGS. These genetic variants were distributed over all possible mutation classes and across tumour types underpinning the importance of comprehensive genomic tumour profiling for precision medicine. In another study from the same consortium, it was shown that more than 30% of patients who received such off-label treatment showed clinical benefit across a diversity of targeted treatments [2].

Whilst the potential of WGS as a comprehensive diagnostic tool has been demonstrated in a number of studies [1, 3–6], its feasibility in a routine diagnostic setting has not yet been demonstrated. The WIDE (WGS Implementation in standard Diagnostics for Every cancer patient) study therefore aims to investigate feasibility of WGS-based diagnostics in routine practice and aspects such as clinical validity, cost-effectiveness, added value and the contribution of WGS and clinical data to a centralized database for facilitating cancer research and improving care for future patients [7]. Importantly, WIDE adopts the approach of periodically (i.e. after every 200 patients) evaluating key performance indicators and optimizing procedures accordingly in order to achieve continuous improvement of the performance of WGS in a routine clinical setting.

Here, we describe the conceptual design and experimental conditions for this study.

## Objectives

### Primary objectives

**Table:** 

Primary study endpoint	Primary outcome measure
Feasibility	Percentage of patients for whom processing from biopsy to WGS report is successful and the turnaround time (TAT) of biopsy until WGS report in working days
Clinical validation	Percentage of concordant variants between WGS and SOC molecular based diagnostics

### Secondary objectives

**Table:** 

Secondary study endpoint	Secondary outcome measure
Additional treatment options	Percentage of patients for whom potential treatment options (in clinical trials in the Netherlands) are identified by WGS, which have not been identified with SOC diagnostics
Health Technology Assessment	Costs and benefits associated with WGS and SOC diagnostics
Better informed decision making and experience of the treating clinician	Opinion of treating clinicians on the added value of WGS for clinical decision making compared to SOC diagnostics evaluated through questionnaires
Expand HMF database	The number of patients for whom clinical and WGS data are added to the HMF database for biomarker discovery in cancer research

## Methods

### Study design

WIDE is a prospective observational diagnostic study in the Netherlands Cancer Institute (NKI) which aims to include 1200 patients with metastasized cancer in a time frame of 18–24 months. The study has been approved by the Medical Ethical Committee of the Netherlands Cancer Institute. WIDE is a collaboration between NKI (a comprehensive cancer cancer), the Hartwig Medical Foundation (HMF, a non-profit organisation), and the Utrecht UMC (UMCU, an academic medical hospital).

### Study population

1200 consecutive patients from the Netherlands Cancer Institute will be recruited from patients with (a suspicion) of stage IV disease of solid tumours who are treated at the NKI without pre-selection on tumour type. Patients are eligible when a biopsy or resection material can be safely obtained during a routine diagnostic procedure. Once a SOC biopsy procedure (4 biopsies if possible) has been performed, the tumour material is used for both SOC diagnostics and WGS analysis. Patients of whom a fresh frozen tumour sample has been obtained earlier (either at the NKI or elsewhere), i.e. archival fresh frozen material, are also eligible when they will be treated at the NKI and routine molecular diagnostic analysis on the archival tumour material is requested. Use of archival fresh frozen material is excluded in patients who have received tyrosine kinase inhibitors after the retrieval of tumour material as such treatments may shift the genomic profile by clonal selection. Patients who underwent allogeneic stem cell transplantation, or transplantation of the organ from which the tumour originated or is located, are excluded as well, because matching tumour and blood DNA is required for identifying a full tumour-specific mutational profile. For each WIDE patient no more than one successful WGS analysis will be obtained, except when molecular analysis is indicated in the context of resistance mechanisms which can be relevant for subsequent treatment options e.g. tyrosine kinase inhibitors in lung cancer. If so, patients can have multiple tumour samples analysed by WGS. Patients must be 18 years or older and willing and able to comply with the protocol as judged by the investigator, as well as sign a written consent.

### Study workflow

**Fig. 1:**
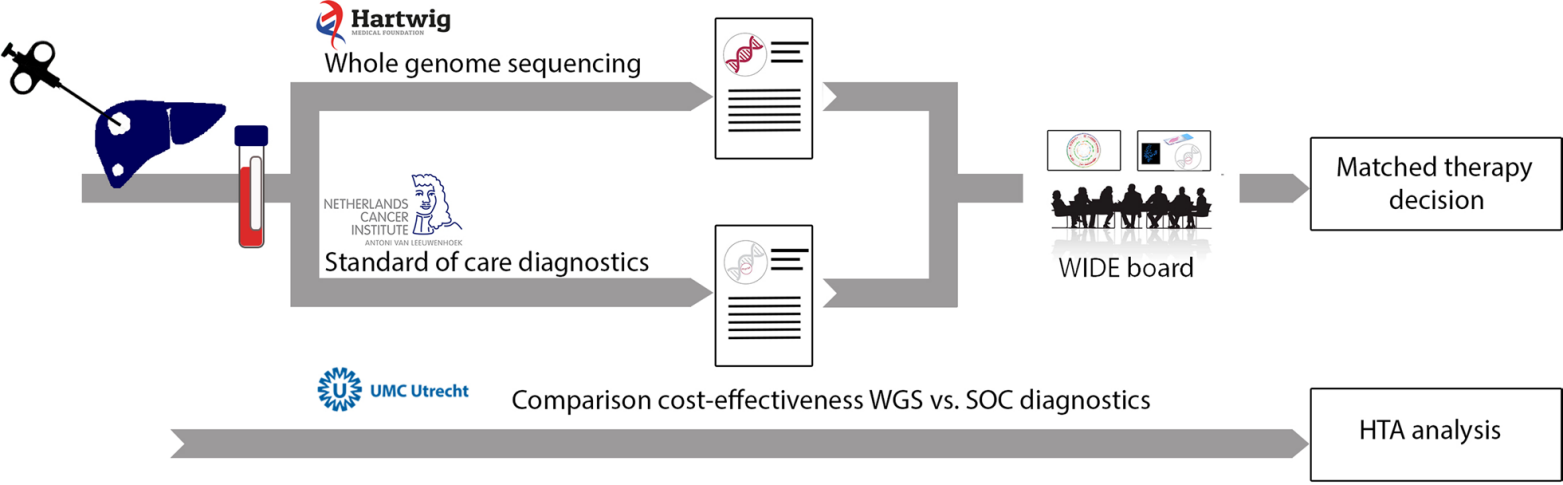
Workflow of the WIDE study. Patients with (a suspicion) of stage IV cancer undergoing a tumour biopsy as part of the routine standard of care at NKI are eligible for inclusion in the WIDE study. In addition, a blood sample is drawn. Subsequently, both a fresh frozen tumour and a blood sample are shipped to HMF for WGS analysis, and a tumour sample will be assessed according to standard of care (SOC). Both the results from WGS and SOC are discussed in a dedicated molecular tumour board and reported for clinical decision making. Alongside, a cost-effectiveness comparison of WGS versus SOC diagnostics will be performed

WGS will be performed prospectively in parallel with and independently of SOC diagnostics, in which SOC diagnostics may or may not include molecular diagnostics (Fig. 1).

### Sample collection

A fresh sample of the primary or metastatic tumour will be obtained as part of routine standard of care (SOC) diagnostic procedures. This can be achieved by means of a needle biopsy, resection or collection of body fluids containing tumour cells, e.g. pleural effusion or ascites, all performed in the context of SOC. If possible, routine SOC diagnostic procedures usually involve multiple tumour biopsies (1–4 biopsies), depending on safety and risk of complications. The majority of biopsy procedures are image guided (e.g. CT or ultrasound). In addition, a 10 ml whole blood sample will be obtained to isolate normal DNA in order to be able to discriminate somatic mutations from the patient’s germline DNA background variations.

### Sample processing

A pathologist determines, depending on the amount of tumour material and the clinical question, whether frozen sections are cut from the biopsies or resection specimens. In case of insufficient tumour material, no frozen sections are cut and all material is used for standard diagnostics. If frozen sections are cut, a pathologist subsequently assesses the tumour cell percentage (TCP). In case of multiple biopsies, a pathologist decides based on the TCP which biopsy is most suitable for standard diagnostics (depending on the clinical question) and which biopsy for WGS analysis. Subsequently, the tumour area is marked for manual macrodissection when needed. For body fluids (e.g. pleural effusion or ascites) a TCP of 30% or higher is considered sufficient for WGS. For tissue samples (i.e. biopsies or resections), a TCP of 20% or higher is considered sufficient. When these conditions are met, the frozen tumour sample and a tube of blood are shipped to HMF within 24 h by courier. The biopsy designated for standard diagnostics and leftover biopsy material from macrodissection is processed and embedded in paraffin (formalin fixed paraffin embedded (FFPE)) according to standard procedures. The NKI department of pathology operates under ISO15189 accreditation.

### WGS and bioinformatics

Whole genome DNA sequencing is performed according to standard procedures described previously [1]. All procedures are automated as much as possible and the Illumina® NovaSeqX and NovaSeq6000 platforms are used. First, shallow whole genome sequencing is used to determine an accurate tumour purity of the samples before continuing full sequencing at a sequencing depth of > 90×. DNA isolated from blood is sequenced at depth of > 30× as a germline control. Sequencing data is analysed with an optimized in-house bioinformatic pipeline designed to detect all types of somatic alterations, including single and multiple nucleotide substitutions (SNV and MNV), insertions and deletions (indels), copy number alterations (amplifications and gene copy losses) and genomic rearrangements and structural variants (e.g. gene fusions) [8]. Results from the tumour and germline sample are compared to filter out germline polymorphisms, which enables the reporting of somatic variants and therapeutically actionable germline variants (as described below) only. All other germline variations are automatically subtracted from the somatic mutations in the bioinformatics analysis and are not disclosed to the investigators, nor are they reported. All code and scripts used for analysis of the WGS data are publicly available via Github [9]. HMF has established procedures for WGS under ISO17025 accreditation.

### SOC molecular diagnostics

The standard of care molecular diagnostics portfolio at NKI comprises of targeted next generation sequencing (NGS) panel (Ampliseq, Cancer hotspot panel V2, Illumina Inc, San Deigo, United States of America), RNA-based NGS fusion analysis (Archer Fusionplex, Lung and Sarcoma panels, Archer DX Inc, Boulders, United States of America), Sanger sequencing, reverse transcriptase polymerase chain reaction (RT-PCR), Multiplex fragment analysis polymerase chain reaction, High Resolution Melting (HRM), fluorescence in situ hybridization (FISH) and immunohistochemistry (IHC), respectively.

### Reporting

A detailed molecular patient report (OncoAct, Hartwig Medical Foundation, Amsterdam, the Netherlands) is produced with all variants that are relevant for diagnostic purposes and cancer treatment decision making (actionable variants), including both somatic and germline variants. Upon patient preference declared in the informed consent, selected germline variants are being reported back as inherited variants, along with an offer for routine clinical genetics counselling. Moreover, actionable mutations are linked to existing biomarker-based clinical studies. In case a reported variant is identified by WGS but not in SOC or vice versa, additional verification tests will be performed in order to resolve the cause of the discordance. WGS and SOC results from all patients are discussed in a weekly WIDE-dedicated molecular tumour board, consisting of clinical molecular biologists, pathologists, clinical geneticists and medical oncologists. Ultimately, according to standard reporting procedures of the NKI, WGS results are included in the pathology report and included in the electronic patient record (Hix, Chipsoft, Amsterdam, The Netherlands) as well as the Dutch national pathology digital archive (PALGA) [10]. Results from both SOC and WGS diagnostics are used for clinical decision making and can be discussed if requested by the treating clinician in a molecular tumour board (MTB). Treatment decisions will be made based on the expertise of these multiple disciplines. In case of any persistent discordant results between WGS and SOC diagnostics, SOC findings will be leading in treatment decision making. Actionable variants identified by WGS can therefore potentially result in adjusting the (initial) treatment plan. The number of patients included in (experimental) therapies initiated based on SOC or WGS diagnostics will be reported with at least 3 months follow up. The clinical efficacy of (experimental) therapies is beyond the scope of WIDE.

## Primary endpoints

### Feasibility of WGS in routine clinical practice

In order to measure the feasibility of WGS in routine clinical practice, the percentage of patients for whom processing from biopsy to WGS report is successful and the turnaround time (TAT) of biopsy until WGS report in working days, will be determined.

### Clinical validation

For clinical validation, the percentage of variants for which WGS detects (at minimum) the same treatment-relevant variants as DNA-based SOC tests, will be reported. Variants considered include Single Nucleotide Variants (SNVs), Multi Nucleotide Variants (MNVs), Insertions/Deletions (INDELs), Copy Number Alterations (CNAs), Structural Variants (SVs) and other tumour characteristics (e.g. MSI status). For this endpoint, only those variants which are detectable with SOC will be evaluated.

## Secondary endpoints

### Health technology assessment

For the HTA, micro-costing and budget impact analysis of WGS compared to SOC diagnostics will be performed, based on the Activity Based Costing Method. The HTA will include the costs of personnel, turnaround time (TAT), equipment/material, tests and platforms used, potentially iterative sequences of SOC tests, consequences of treatment decisions made etc. compared to WGS. In order to extrapolate findings nation-wide, the global International Society for Pharmacoeconomics and Outcomes Research (ISPOR) guidelines will be used.

### Additional treatment options

For the endpoint additional treatment options, the percentage of patients for whom treatment-relevant variants identified by WGS and not by SOC diagnostics, for which on-label or off-label drugs exist, will be reported and details will be described.

### Better informed clinical decision making

The opinion of treating clinicians on the added value of WGS in terms of clinical decision making compared to SOC diagnostics will be collected through digital questionnaires (qualitative analysis) which are developed for this study and provided as Additional files 1 (questionnaire at start of study), 2 (questionnaire halfway) and 3 (questionnaire at end of study). In addition, feedback of clinicians is collected by performing in depth interviews in order to ensure that the WGS report format meets their needs. If treating clinicians experience barriers in acting upon the patient WGS report format, additional educational activities will be arranged and/or the report format will be adapted accordingly.

### Enriching the HMF database

In a learning healthcare system, it is pivotal to generate as much as possible potentially informative real world data, in this case clinically well annotated WGS results of metastatic tumours which are made available for future research. Therefore the data generated within the WIDE study will be added to the HMF database (https://www.hartwigmedicalfoundation.nl/data-aanvragen/). This database (currently storing > 4500 patients) contains pseudonomized genetic and clinical data, including treatment and treatment outcomes, of individuals whose tumours have been sequenced by HMF in the context of multiple studies. Variables documented include date of biopsy, biopsy site, sample type, standard of care and WGS characteristics, biomarkers identified, diagnosis, pre-treatment, treatment and radiological or clinical treatment response (predominantly according to Response Evaluation Criteria In Solid Tumours (RECIST) 1.1). Only patients who provide additional consent for re-use of their genomic and (limited) clinical data for cancer research purposes will be added to the HMF database. The HMF database is accessible for international researchers through an access-controlled mechanism. The data access request procedure involves evaluation of scientific, legal and ethical aspects of the intended data usage and applications are assessed by an independent scientific and data access board.

## Statistical analysis

### Sample size calculation

The sample size of 1200 patients has been calculated based on the primary endpoint ‘clinical validation’. The aim is to detect in at least 95% of the cases the same variants as SOC molecular tests. For the lower limit of the confidence interval to be at least 95% and under the assumption that the real concordance rate will be 97.5%, we will have a power of 96% to find the lower limit of the confidence interval to be at least 95% when we include 624 individual detected variants. Assuming that 30% of biopsies do not have detectable genomic aberrations in SOC, a total of 1200 patients need to be included in this study.

### Continuous evaluation and improvement

In the WIDE study, the primary and secondary endpoints are evaluated periodically after every 200 patients. Based on these interim analyses, according to the PDCA approach (plan-do-check-act) and in line with ISO 151890 principles, procedures are being optimized and effects monitored, aiming to achieve continuous improvement of WGS implementation in a routine clinical setting. After completing the procedure for every 200 patients, feasibility, clinical validation and added value are systematically evaluated by descriptive statistics. In order to ensure that the sample size for the endpoint clinical validation is reached and the added value is safeguarded, there are multiple strategies in place. If the number of patients for whom SOC molecular diagnostics is performed is < 70%, the inclusion strategy of the treating physicians will be discussed and adjusted to select more patients for whom routine molecular diagnostics is indicated. If for any given tumour type the added value is less than 10%, the inclusion criteria will be adjusted (e.g. excluding tumour types for which no additional targets are being found). At the start, halfway (upon inclusion of 600 patients) and at the end of the WIDE study, the (expectations of the) clinical value of WGS according to treating clinicians will be evaluated by means of a digital survey. In case less than 50% of the treating clinicians indicate WGS has added value compared to SOC diagnostics, inclusion criteria will be reconsidered. Similarly halfway, the patient report format will be evaluated based on comprehensibility and usefulness of the provided information. We aim to hereby identify important barriers and facilitators for integrating WGS-based diagnostics in clinical practice.

## Discussion

In the WIDE study, for the first time the feasibility and impact of WGS-based diagnostics in routine clinical practice will be systematically investigated in a prospective setting. The WIDE study embeds WGS, with its unprecedented opportunities to match the specific biology of a patient’s tumour with the right drug, in a routine pathology diagnostic workflow, thereby allowing streamlining of patient care and laboratory logistics. In patient care, treating clinicians play a crucial role in the implementation of WGS and therefore they will receive dedicated training on interpretation of the patient reports. Moreover, all patient reports based on WGS data as well as the results of SOC diagnostics will be discussed in a  WIDE-dedicated MTB. Regarding laboratory logistics at HMF, experimental, bioinformatics and reporting procedures will be optimized for use in routine diagnostics (including reduction of TAT to 12 working days). Similarly, laboratory logistics at the NKI will be adjusted to accommodate WGS diagnostics. Importantly, logistics that can work with fresh or fresh-frozen samples (beyond standard frozen section diagnostics) rather than FFPE (formalin fixed paraffin embedded) need to be incorporated in the routine diagnostic workflow. Formalin fixation fragments DNA and causes artefacts, which interfere with genome-wide mutation and structural variant calling. Moreover, fixation renders a substantial amount of DNA unusable by which precious tumour material is lost. As a consequence, frozen sections need to be cut from every tumour sample for sample input evaluation. Since a tumour cell percentage of > 20% is a prerequisite for successful and cost-effective WGS analysis (at 90/100 × sequencing depth), the tumour cell percentage needs to be assessed first by a pathologist before shipping to HMF. As a result of the continuous evaluation and improvement approach, diagnostic and treatment logistics will frequently be adapted. As such this study will not only investigate feasibility, clinical value, added value and cost-effectiveness, but also identify a sound generic implementation strategy for WGS in routine clinical practice which can be adopted by other interested hospitals. Consequently, this study will generate new insights in the barriers and facilitators that can be encountered when WGS is integrated in routine clinical care and form the basis of a generic implementation strategy. Moreover, WGS data will be generated of 1200 metastasized solid tumours, which will shed more light on tumour molecular biology and options for targeted therapy, particularly in tumour types for which molecular diagnostics is not yet part of standard of care. The WGS and clinical data will be added to the HMF database which provides extensive opportunities for future research.

## Supplementary information

**Additional file 1:** Questionnaire at start of study.

**Additional file 2:** Questionnaire halfway.

**Additional file 3:** Questionnaire at end of study.

## Data Availability

The datasets generated during the current study are available from the corresponding author on reasonable request.

## Abbreviations

WIDE: WGS Implementation in standard Diagnostics for Every cancer patient
SOC: Standard of care
WGS: Whole genome sequencing
NKI: Netherlands Cancer Institute
HTA: Health technology assessment
NGS: Next generation sequencing
RT-PCR: Reverse transcription polymerase chain reaction
HRM: High Resolution Melting
FISH: Fluorescence in situ hybridization
IHC: Immunohistochemistry
HMF: Hartwig Medical Foundation
CPCT: Center for Personalized Cancer Treatment
TAT: Turnaround time
SNVs: Single Nucleotide Variants
MNVs: Multi Nucleotide Variants
INDELs: Insertions/Deletions
CNAs: Copy Number Alterations
SVs: Structural Variants
MSI: Microsatellite instability
TMB: Tumour mutational burden
TCP: Tumour cell percentage
MTB: Molecular tumour board
ISPOR: International Society for Pharmacoeconomics and Outcomes Research
RECIST: Response Evaluation Criteria In Solid Tumours
PDCA: Plan-do-check-act
FFPE: Formalin fixed paraffin embedded
