# Supplementary material for: Study protocol: Whole genome sequencing Implementation in standard Diagnostics for Every cancer patient (WIDE)
Source: BMC Med Genomics. 2020 Nov 10;13:169. doi: 10.1186/s12920-020-00814-w (PMC7654005; doi:10.1186/s12920-020-00814-w)
Supplement: Supplementary file 2 — Additional file 2: Questionnaire halfway. [file 12920_2020_814_MOESM2_ESM.docx]

**WIDE project - Survey clinicians – Midterm**

**Introduction**

As part of the WIDE-project, we would like to (in terms of a qualitative analysis) evaluate how treating clinicians experience the value of molecular diagnostic (reporting) based on Whole Genome Sequencing (WGS) with regards to clinical decision making compared to the current reporting of standard of care (SOC) molecular diagnostics. This survey will therefore be performed at the start, midterm and at the end of the WIDE-project based on the questionnaire below. The questionnaires are anonymous. The first survey you have filled out at the start of the project. This is the second survey. The last survey will follow at the end of the project (after 1 year). You will receive an automatic message as a reminder.

We want to kindly request you to fill out this first survey *within 2 weeks*.

Filling out the questionnaire will take 5 minutes at maximum.

On behalf of the entire team of the WIDE-project, we want to thank you in advance for your contribution!

**Explanatory word list:**

- *WGS* = Whole Genome Sequencing
- *SOC:* standard molecular diagnostics (for example targeted panel sequencing, Fluorescent in Situ Hybridization (FISH), immunohistochemistry (IHC))

**Part 1 – Multiple choice and open questions**

You can find several multiple choice and open questions below. You only need to fill out one answer, unless otherwise is stated.

1. **What type of clinician are you?**

- Medical oncologist
- Pulmonologist
- Neurologist
- Surgeon
- Radiotherapist
- Otherwise ……………………………………………………………………………………..

1. **Are you content with the current offer of standard of care (SOC) molecular diagnostics?**

- Yes
- No

1. **Are you missing anything in the current SOC (in terms of content or reporting)?**

- Yes
- No

1. **If so, can you specify what you are missing in the current SOC (in terms of content or reporting?** *(Multiple answers are possible).*

- Reporting of all findings in one comprehensive report
- Feedback when the usual turnaround time is exceeded
- Information on loss of heterozygosity (LOH)
- Information on (treatable) germline variants
- Information on tumour mutational load / tumour mutational burden
- Information on ‘gene disruptions‘ (for example inversions, deletions, insertions, etc.)
- Fast implementation of new biomarkers
- Identification of new therapeutic opportunities (off label / clinical trials)
- Otherwise, ……………………………………………………………………………………………

1. **Do you think molecular diagnostics by means of WGS has added value for clinical decision making, compared to SOC?**

- Yes
- No

1. **If so, can you indicate what the added value would be for you (in terms of content and reporting)? (***Multiple answers are possible.)*

- All genomic information and potential actionable targets are summarized in *one report*
- I can immediately apply all new biomarkers clinically (without the need for validation of a new technique)
- It can help me identify new therapeutic possibilities for patients for whom regular therapeutic options are no longer available
- I receive information on tumour mutational load/ tumour mutational burden
- I receive information on gene disruptions (for example inversions, deletions, insertions)
- I receive information on gene amplifications and deletions
- I receive information on treatable germline variants
- I receive information on loss of heterozygosity (LOH).
- Otherwise, ……………………………………………………………………………………………………………
- Not applicable

1. **If no, can you indicate why you think that WGS will have no added value for you (in terms of content and reporting)?** *(Multiple answers are possible).*

- I think the turn around time from biopsy to result takes too long
- I think the extensive report based on WGS information is too complex to interpret
- I receive too much information, also information of which the clinical relevance is unknown to me (for example ‘variants of unknown significance’)
- I think the WGS information is not presented clear enough
- I don’t use new biomarkers in general (as long as these are not adopted into the guidelines)
- Otherwise, ………………………………………………………………………………………………………………
- Not applicable

1. **To what extent according to you does the clinical utility of a new biomarker (found with WGS) has to be proven, prior to implementation into routine practice?**

- At least with evidence of preclinical research
- At least with evidence of retrospective clinical research
- At least with evidence of prospective/observational clinical research
- At least with evidence of randomized clinical research
- Otherwise,……………………………………………………………………………………………………….

1. **To what extent you think that *the profession* needs the clinical utility of a new biomarker (found with WGS) to be proven, in order for the biomarker to be adopted in the guidelines?**

- At least with evidence of preclinical research
- At least with evidence of retrospective clinical research
- At least with evidence of prospective/observational clinical research
- At least with evidence of randomized clinical research
- Otherwise,……………………………………………………………………………………………………….

1. **Do you think a cost-effectiveness analysis with regards to WGS will be of added value?**

- I’m neutral
- I think this information is important for the adoption of WGS in case of relevant medical indications in the basic insurance package
- I think this has no added value, to prove clinical utility is sufficient
- Otherwise, ……………………………………………………………………………………………………………

1. **How many WGS reports have you received in the last months from the WIDE project?**

- 0 – 5
- 5 – 15
- 15 – 30
- > 30

1. **Has the WGS report aided you to identify new therapeutic possibilities for patients for whom regular treatment options were no longer available (either off-label, or clinical trials)?**

- No, never
- Yes, for about *10%* of my patients who participated
- Yes, for about *25%* of my patients who participated
- Yes, for *half* of my patients who participated
- Yes, for the *majority* of my patients who participated
- Yes, for *all* of my patients who participated

1. **Have you used the WGS report to immediately apply new biomarkers into clinical practice?**

- Yes
- No

1. **Did you find it difficult to independently interpret the WGS report?**

- Yes
- No

If so, can you indicate what needs to be improved to make it easier for you to interpret the WGS report?

………………………………………………………………………………………………………………………………………………………………………………………………………………………………………………………………………………………………

**Part 2 – Statements**

The statements below concern the added value in terms of content and reporting of molecular diagnostics based on WGS compared to SOC.

Indicate below to what extent you agree with the following statements:

**1 = I very much disagree, 2 = I disagree, 3 = neutral, 4 = I agree, 5 = I very much agree**

1. The turn aorund time from biopsy to results of WGS has to be within 2 weeks

2

3

4

5

1

1. I think the WGS report is mostly too complex to independently interpret

2

3

4

5

1

1. I think the WGS report is mostly too complex to explain to my patient

2

3

4

5

1

1. I have insufficient time to discuss the full WGS report with my patient

2

3

4

5

1

1. I do not know how to deal with possible (germline) incidental findings

2

3

4

5

1

(for example BRCA 1 / 2 mutations, MSI of nom-oncological

findings)

1. I prefer WGS over SOC because all possible molecular diagnostic tests are combined in one, therefore I receive one *report* in which all results are summarized

2

3

4

5

1

1. I prefer WGS over SOC because new biomarkers can be immediately applied clinically (without the need for validation of a new technique)

2

3

4

5

1

1. The WGS rapport has helped me identify new therapeutic possibilities for patients for whom regular therapeutic options are no longer available (either off-label, or in clinical trials)

2

3

4

5

1

1. WGS has *no* added value for me in my current clinical decision making

2

3

4

5

1

(as compared to SOC)

**Part 3 – Additional comments/reactions**

Below there are three last open questions.

1. **If there was no barrier whatsoever to use WGS, *when* and *for which* indications would you use WGS?**

………………………………………………………………………………………………………………………………………………………………………………………………………………………………………………………………………………………………………………………………………………………………………………………………………………………………………………

1. **Do you have additional comments or suggestions concerning the WIDE project you would like to share?**

………………………………………………………………………………………………………………………………………………………………………………………………………………………………………………………………………………………………………………………………………………………………………………………………………………………………………………

1. **Are there questions or statements you have missed in this questionnaire?**

……………………………………………………………………………………………………………………………………………………………………………………………………………………………………………………………………………………………………………………………………………………………………………………………………………………………………………...
